# Resolvin D2 Reduces Chronic Neuropathic Pain and Bone Cancer Pain via Spinal Inhibition of IL-17 Secretion, CXCL1 Release and Astrocyte Activation in Mice

**DOI:** 10.3390/brainsci13010152

**Published:** 2023-01-15

**Authors:** Jun Pang, Pengfei Xin, Ying Kong, Zhe Wang, Xiaopeng Wang

**Affiliations:** 1 Department of Anesthesiology & Center for Brain Science, the First Affiliated Hospital of Xi’an Jiaotong University, Xi’an 710061, China; 2 Department of Anesthesiology, Shanxi Bethune Hospital, Shanxi Academy of Medical Sciences, Tongji Shanxi Hospital, Third Hospital of Shanxi Medical University, Taiyuan 030032, China; 3 Tongji Hospital, Tongji Medical College, Huazhong University of Science and Technology, Wuhan 430030, China; 4 Department of Stomatology, Shanxi Bethune Hospital, Shanxi Academy of Medical Sciences, Tongji Shanxi Hospital, Third Hospital of Shanxi Medical University, Taiyuan 030032, China

**Keywords:** RvD2, neuropathic pain, bone cancer pain, IL-17, CXCL1, astrocyte activation

## Abstract

Chronic pain burdens patients and healthcare systems worldwide. Pain control remains urgently required. IL-17 (interleukin-17)-mediated neuroinflammation is of unique importance in spinal nociceptive transduction in pathological pain development. Recently, resolvin D2 (RvD2), as a bioactive, specialized pro-resolving mediator derived from docosahexaenoic acid, exhibits potent resolution of inflammation in several neurological disorders. This preclinical study evaluates the therapeutic potential and underlying targets of RvD2 in two mouse models of chronic pain, including sciatic nerve ligation-caused neuropathic pain and sarcoma-caused bone cancer pain. Herein, we report that repetitive injections of RvD2 (intrathecal, 500 ng) reduce the initiation of mechanical allodynia and heat hyperalgesia following sciatic nerve damage and bone cancer. Single exposure to RvD2 (intrathecal, 500 ng) attenuates the established neuropathic pain and bone cancer pain. Furthermore, systemic RvD2 (intravenous, 5 μg) therapy is effective in attenuating chronic pain behaviors. Strikingly, RvD2 treatment suppresses spinal IL-17 overexpression, chemokine CXCL1 release and astrocyte activation in mice undergoing sciatic nerve trauma and bone cancer. Pharmacological neutralization of IL-17 ameliorates chronic neuropathic pain and persistent bone cancer pain, as well as reducing spinal CXCL1 release. Recombinant IL-17-evoked acute pain behaviors and spinal CXCL1 release are mitigated after RvD2 administration. In addition, RvD2 treatment dampens exogenous CXCL1-caused transient pain phenotypes. Overall, these current findings identify that RvD2 therapy is effective against the initiation and persistence of long-lasting neuropathic pain and bone cancer pain, which may be through spinal down-modulation of IL-17 secretion, CXCL1 release and astrocyte activation.

## 1. Introduction

Chronic pain is clinically common and often accompanies nerve injuries, arthritis, bone fractures, cancer and chemotherapy, constituting a major burden to individuals and society worldwide [1,2]. Statistically, the incidence of chronic pain is over 30% in America and China, as well as approximately 13–50% in the UK [2,3,4]. Effective treatments for chronic pain continue to be inadequate. Recently, accumulating evidence elucidated the important role of excitatory neuronal plasticity in nociception-coding neurocircuits for central pain sensitization and chronic pain phenotypes [5,6]. However, the exact mechanisms underlying chronic pain perception remain elusive.

It is generally identified that neuroinflammatory responses drive neuronal hyperexcitability and facilitate synaptic transmission in the pathophysiology of chronic pain [7,8,9,10,11]. A cardinal feature of neuroinflammation is the secretion of proinflammatory mediators, the generation of chemokines as well as the activization of glial cells [12]. Astrocytes, which are considered to provide nutrient and structural support for neurons in the brain and spinal cord, have gradually been considered a unique requirement for neuroinflammation and connection with neurons in several neurological disorders [13,14]. In particular, astrogliosis is demonstrated to regulate neural responsiveness in the development of pathological pain with different etiologies, such as peripheral tissue injuries, peripheral nerve damage, spinal cord trauma, cancer, opioid therapies and chemotherapies [7,15]. Furthermore, a robust secretion of IL-17 (interleukin-17) following the activation of astrocytes is induced in rodent animals with inflammatory pain resulting from intraplantar exposure to complete Freund’s adjuvant (CFA) [16]. Pharmacological neutralization of IL-17 is effective against remifentanil-induced hyperalgesia via suppressing astrocyte activation and excitatory synaptic transmission in rats [17]. Additionally, chemokine CXCL1, as a characteristic astrocyte-activating factor localized in astrocytes, involves spinal nerve ligation (SNL)-related neuralgia and paclitaxel-induced peripheral neuropathy via astrogliosis [18,19]. Nevertheless, whether targeting astrocyte-coding neuroinflammation may improve chronic pain needs to be clearly investigated.

Specialized pro-resolving mediators (SPMs), derived from the omega-3 polyunsaturated fatty acids docosahexaenoic acid (DHA) and eicosapentaenoic acid (EPA), are critical in the resolution of inflammatory response in many pathological conditions [20]. Resolvins, belonging to the group of SPMs, are bio-synthesized to facilitate inflammation resolution [21]. Strikingly, exogenous D-series resolvin (resolvin D1 and D5) injections exhibit potent anti-inflammatory and anti-nociceptive properties in inflammatory hyperalgesia, neuropathic allodynia and fracture-associated postoperative pain [22,23,24,25]. Although D-series resolvins have been tested in different pathological pain conditions, the involvement of resolvin D2 (RvD2) in chronic pain relief is unexplored.

Herein, we characterized the therapeutic potential of intrathecal (i.t.) and intravenous (i.v.) RvD2 in chronic pain using two mouse models of sciatic nerve injury-caused neuropathic pain and sarcoma-caused bone cancer pain. Expressions of spinal IL-17, CXCL1 and glial fibrillary acidic protein (GFAP) were evaluated to indicate the activation of astrocyte as pivotal drivers of persistent pain and verify analgesic targets of RvD2. Our findings suggest that mitigation of neuroinflammation by RvD2 therapy may be a promising neurotherapeutic avenue to improve chronic pain states.

## 2. Materials and Methods

### 2.1. Animals

Adult male C57BL/6J mice (8–10 weeks old) were randomly housed in appropriate cages under a 12 h dark-light environment and provided water and food ad libitum. The experimental animal center of the Chinese Academy of Military Medical Science provided all animals for the current study. All experimental procedures were performed in rigorous accordance with the International Association for the Study of Pain directives. Ethical permission for the study was obtained by the Laboratory Animal Ethical and Welfare Committee of Shanxi Bethune Hospital at the Shanxi Academy of Medical Science (Shanxi, China).

### 2.2. Drugs and Administration

RvD2 (MedChemExpress, Shanghai, China) was dissolved in 2% ethanol (vehicle) for i.t. and i.v. injection. Recombinant CXCL1 (Abcam, Cambridge, UK), recombinant IL-17 (ProSpec-Tany TechnoGene Ltd., Rehovot, Israel) and a neutralizing antibody against IL-17 (anti-IL-17, Santa Cruz Biotechnology, Santa Cruz, CA, USA) were dissolved in a vehicle (10% DMSO) for i.t. administration. We selected all the dosages of drugs based on the published literature [18,26,27]. The intrathecal injection was performed in sevoflurane (induction = 3.0%; maintenance = 1.5%; Maruishi Pharmaceutical Co., Ltd., Osaka, Japan) anesthetized mice, and drugs were administered by a lumbar puncture between the L_4_ and L_5_ vertebrae through a 30 G needle [28], while 5 μL of the reagent was delivered following detection of a reflexive tail flick.

### 2.3. Neuropathic Pain Model

A mouse model of chronic constriction injury (CCI) of the sciatic nerves, formed as in the published protocols [29], was utilized for causing peripheral nerve trauma. Specifically, the mice were anesthetized with sevoflurane (induction, 3.0%; surgeries, 1.5%) by a nose mask under sterile conditions. The left common sciatic nerve was exposed at the mid-thigh level. The area proximal to the sciatic trifurcation was freed of adhering tissue, with 4 loose ligatures around the nerve using 4–0 braided silk thread at an interval of approximately 1 mm. Sham procedures were made identically with no ligation of the sciatic nerves.

### 2.4. Bone Cancer Pain Model

For the generation of bone cancer pain, 2472 NCTC murine sarcoma cells (2 × 10^5^, 5 μL, American Type Culture Collection, Manassas, VA, USA) were injected into the distal femur condyle according to the published literature [27]. Animals receiving one injection of the identical volume of the culture solution (vehicle) served as controls.

### 2.5. Behavioral Testing

All tests were conducted between 10:00 am and 3:00 pm on that day in a temperature-controlled room at 24 °C. The baseline threshold was tested 1 day before the treatment, and the mice were habituated 2 h per day in the testing apparatus for 3 days prior to the baseline threshold test.

In the von Frey test, the mice were placed on a test platform with a grid spacing of 1.5 mm, covered with a plexiglass box of 49 × 33 × 40 cm and allowed to acclimatize for 2 h. The paw withdrawal threshold (PWT) of the mice was measured with von Frey filaments (Stoelting, Wood Dale, IL, USA) between 0.16 and 2 g using the up-and-down method. Starting with 0.16 g to stimulate the left hind paw, licking or withdrawal during the 5 s stimulus was considered a positive response. The force was reduced in the case of a positive reaction; otherwise, the force was increased, and finally the PWT was calculated [30].

Thermal sensitivity was determined using a hot plate (YLS-6B, Huaibei Zhenghua, Biological Instrument Equipment Co., Ltd., Anhui, China), which was consistent with previous studies [31], and documented as the paw withdrawal latency (PWL). The time the mice spent on the heated surface (52 °C) before showing signs of nociception, including clear paw withdrawal, shaking and licking, was recorded.

The data of the behavioral testing were collected by the same investigator blinded to the grouping as well as the treatments.

### 2.6. ELISA Analysis

An enzyme-linked immunosorbent assay (ELISA) was used to measure the concentrations of CXCL1 (Abcam, ab216951), IL-17 (Abcam, ab100702) and GFAP (Abcam, ab233621) in the L4–5 segments of the spinal cord. Spinal cord tissues were homogenized in a lysis buffer containing protease and phosphatase inhibitors. Tissue samples were centrifuged at 12,500× *g* for 10 min, and the supernatant was collected. A BCA Protein Assay (Pierce, Rockford, IL, USA) was employed to determine the protein concentrations. For each reaction in a 96 well plate, 100 μg of proteins of the samples was used. All ELISA experiments followed the manufacturer’s protocol. The optical densities of the samples were measured using an ELISA plate reader (Bio-Rad, Hercules, CA, USA) at a wavelength of 450 nm, and the levels of CXCL1, IL-17 and GFAP were calculated using the standard curves and normalized to the total protein levels.

### 2.7. Immunofluorescence

The mice were deeply anesthetized and transcardially perfused with pre-cooled PBS following 4% paraformaldehyde. The whole spinal cord was blown out using the hydraulic pressure method. The L_4_–L_5_ spinal cord was dissected and dehydrated in 30% sucrose for 2 days. The tissues were then frozen in O.C.T. and cut into 8 µm frozen sections using a cryostat (Leica Biosystems, Heidelberg, Germany). The sections were blocked with 0.3% Triton X-100 for 10 min and 5% goat serum for 1 h. They were then incubated with the primary antibodies overnight at 4 °C. The following primary antibody was used: anti-GFAP (1:200, Abcam). After rinsing three times with PBS, the sections were incubated with a fluorescence-labeled secondary antibody for 1 h. Images were collected using a fluorescence microscope (Olympus, Tokyo, Japan), and the analysis was performed using Image J software.

### 2.8. Statistical Analysis

All statistical analyses were carried out using SPSS 18.0 software (SPSS, Chicago, IL, USA). All animals were randomly assigned to experimental conditions. We calculated the sample size based on previous reports in pain research [30,31]. Results were expressed as the mean ± SEM. The difference in the behavioral data was compared using two-way analysis of variance (ANOVA) followed by Bonferroni post hoc multiple comparisons. The difference in biochemical results was detected using one-way ANOVA with Bonferroni multiple comparisons. Statistical significance was established at *p* < 0.05.

## 3. Results

### 3.1. Intrathecal Administration of RvD2 Prevents and Alleviates Persistent Neuropathic Pain following Peripheral Nerve Trauma

**Figure 1:**
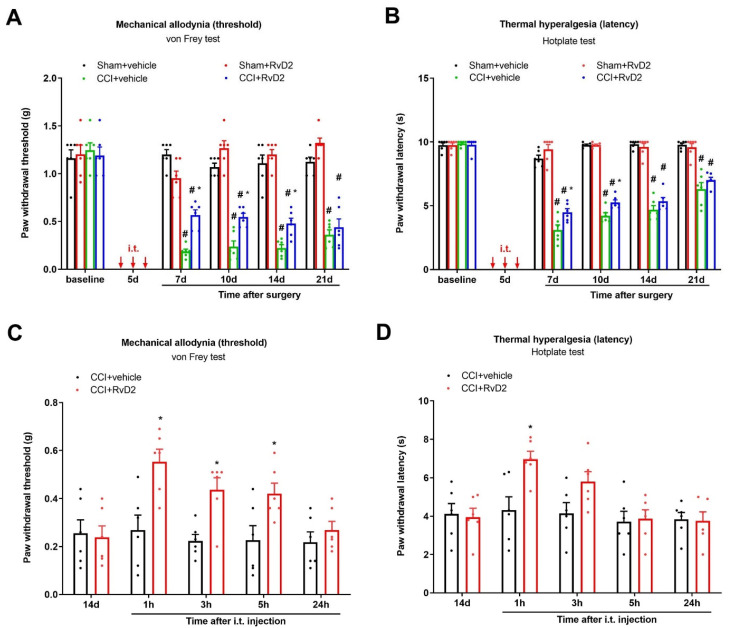
Intrathecal treatment with RvD2 reduces the production and persistence of neuropathic pain caused by peripheral nerves injuries. (**A**,**B**) Intrathecal RvD2 (500 ng) was given daily on days 4, 5 and 6 (indicated by red arrows) following sham and CCI operations. (**C**,**D**) RvD2 (i.t., 500 ng) was given on day 14 following CCI surgeries. Mechanical allodynia was evaluated by paw withdrawal threshold (**A**,**B**) using von Frey experiments, and thermal hyperalgesia was evaluated by paw withdrawal latency (**B**,**D**) using hotplate experiments. Behavioral results are expressed as the mean ± SEM (*n* = 6) and compared using two-way ANOVA followed by Bonferroni multiple comparisons. # *p* < 0.05 vs. group sham + vehicle. * *p* < 0.05 vs. group CCI + vehicle. i.t. = intrathecal injection.

Initially, there was no difference in basal mechanical sensitivity (PWT) or thermal sensitivity (PWL) among the groups when using the von Frey test and hotplate test (Figure 1A,B). Compared with the baseline, the PWT and PWL did not change after the sham surgeries, suggesting that the sham surgery did not induce a pain phenotype (Figure 1A,B). As expected, the CCI surgeries caused a long-lasting decrease in the PWT and PWL, suggesting the development of chronic neuropathic pain (thermal hyperalgesia and mechanical allodynia) following peripheral nerve trauma (*p* < 0.05; Figure 1A,B).

Then, to investigate the therapeutic potential of RvD2 in long-lasting neuropathic pain, RvD2 (i.t., 500 ng) was injected daily on days 4, 5 and 6 (in the early phase) following the CCI operation. Intriguingly, repetitive injections of RvD2 reduced the CCI-induced mechanical allodynia and heat hyperalgesia, as characterized by the dramatic rise in the mechanical PWT (F (3, 100) = 152.7, *p* < 0.0001, *n* = 6, two-way ANOVA; Figure 1A) and the considerable extension in the heat PWL (F (3, 100) = 310.8, *p* < 0.0001, *n* = 6, two-way ANOVA; Figure 1B). Such preventive effects of RvD2 on neuropathic pain were strong for at least 4 days following the three injections.

We further tested the efficiency of post-administration of RvD2 in existing pain relief. In the von Frey test, a single delivery of RvD2 (i.t., 500 ng) on day 14 following CCI intervention (in the late phase) exhibited quick and transitory mitigation of the existing mechanical allodynia for 5 h (F (1, 50) = 21.7, *p* < 0.0001, *n* = 6, two-way ANOVA; Figure 1C) in the mice undergoing peripheral nerve injury. As a parallel, in the hotplate test, post-treatment with RvD2 also decreased the existing heat hyperalgesia for 1 h, as reflected by the increase in the thermal PWL in the CCI animals (F (1, 50) = 6.84, *p* = 0.0118, *n* = 6, two-way ANOVA; Figure 1D). Collectively, intrathecal RvD2 therapy confers protection against the initiation and maintenance of CCI-caused neuropathic pain phenotypes.

### 3.2. Intrathecal Pre-Application of RvD2 Down-Modulates Spinal IL-17 and CXCL1 Overexpression and Astrocyte Activation after Peripheral Nerve Trauma

**Figure 2:**
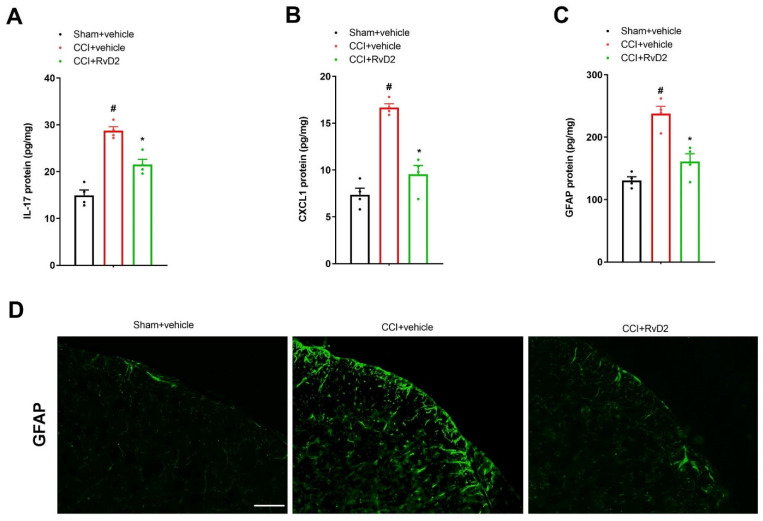
Pretreatment with RvD2 reduced spinal IL-17 secretion, CXCL1 release and astrocyte activation following peripheral nerve trauma. Intrathecal RvD2 (500 ng) was administered daily on days 4, 5 and 6 following CCI operations. All biochemical data were collected on day 7 following sham and CCI interventions. (**A**–**C**) ELISA assays showed that RvD2 pretreatment down-modulated the elevated levels of spinal IL-17, CXCL1 and GFAP (the marker of astrocyte activation) proteins in CCI animals. (**D**) Immunohistochemistry staining showed representative photomicrographs of GFAP in the spinal dorsal horn following CCI surgeries and RvD2 exposure (scale bar, 50 μm). Results from biochemical experiments are presented as the mean ± SEM (*n* = 4) and compared using one-way ANOVA followed by Bonferroni multiple comparisons. # *p* < 0.05 vs. group sham + vehicle. * *p* < 0.05 vs. group CCI + vehicle.

Astrocyte-mediated neuroinflammatory responses in the dorsal horn are of great significance in the pathogenesis of long-lasting pain [8,15]. We employed an ELISA assay to measure the spinal IL-17, CXCL1 and GFAP concentrations. All of the assays reliably produced the expected standard curves of the IL-17, CXCL1 and GFAP concentrations (Supplementary Materials, Figure S1A–C), suggesting reliable measurements within the specified concentration range. We observed an obvious elevation in the spinal expressions of IL-17, CXCL1 and GFAP in the animals 7 days after peripheral nerve trauma using an ELISA assay (*p* < 0.05, *n* = 4; Figure 2A–C). Furthermore, these inflammatory mediators were measured to verify whether they were implicated in RvD2 anti-nociception in the CCI animals. Interestingly, repetitive pre-administration of RvD2 (i.t., 500 ng) suppressed the rise in IL-17, CXCL1 as well as GFAP expression in the dorsal horn following CCI surgeries (*p* < 0.05, *n* = 4; Figure 2A–C). Additionally, we detected the reduction in CCI-induced spinal activation of the astrocytes by RvD2 pretreatment using immunohistochemistry experiments (Figure 2D). Taken together, these biochemical findings suggest that RvD2 prevents long-lasting neuropathic pain through spinal inhibition of neuroinflammation.

### 3.3. Intrathecal Administration of RvD2 Prevents and Alleviates Persistent Bone Cancer Pain

**Figure 3:**
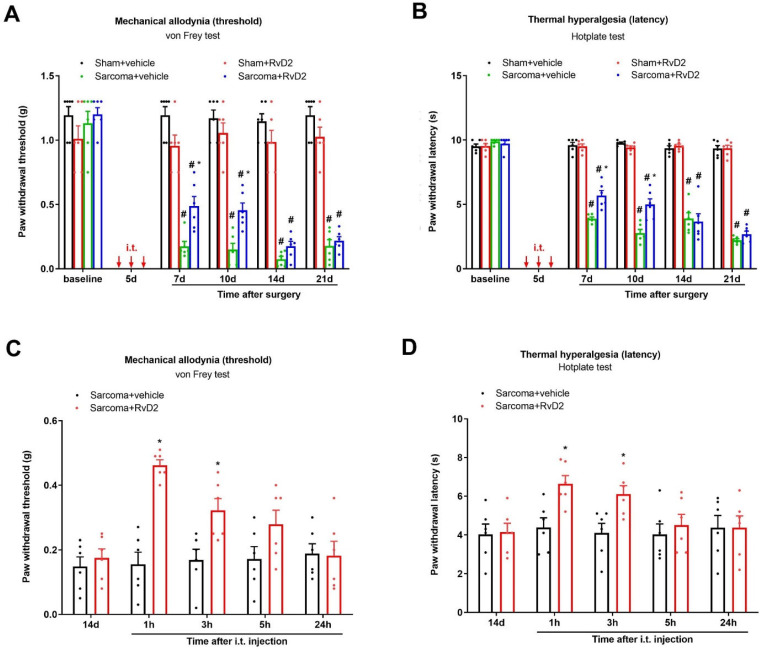
Intrathecal treatment with RvD2 reduced the production and persistence of bone cancer pain following sarcoma cell implantation. (**A**,**B**) Intrathecal (i.t.) RvD2 (500 ng) was given daily on days 4, 5 and 6 (indicated by red arrows) following sham and sarcoma implantations. (**C**,**D**) RvD2 (i.t., 500 ng) was injected on day 14 following sarcoma implantations. Mechanical allodynia was evaluated by paw withdrawal threshold (**A**,**C**) using von Frey experiments, and thermal hyperalgesia was evaluated by paw withdrawal latency (**B**,**D**) using hotplate experiments. Results from behavioral testing are expressed as the mean ± SEM (*n* = 6) and compared using two-way ANOVA followed by Bonferroni multiple comparisons. # *p* < 0.05 vs. group sham + vehicle. * *p* < 0.05 vs. group sarcoma + vehicle.

Next, to evaluate the therapeutic potential of RvD2 in bone cancer pain, repeated injections of RvD2 (i.t., 500 ng) were performed on days 4, 5 and 6 (in the early phase) following the injection of NCTC sarcoma cells into the distal femur condyle. Herein, both the von Frey test and hotplate test detected that sarcoma implantation caused a long-lasting decrease in the mechanical PWT and thermal PWL, suggesting the successful establishment of chronic bone cancer pain (*p* < 0.05; Figure 3A,B). Strikingly, repetitive pretreatment with RvD2 reduced the generation of sarcoma-induced mechanical allodynia and heat hyperalgesia, as indicated by the robust rise in the mechanical PWT (F (3, 100) = 181.6, *p* < 0.0001, *n* = 6, two-way ANOVA; Figure 3A) and the considerable extension in the heat PWL (F (1, 100) = 471.7, *p* < 0.0001, *n* = 6, two-way ANOVA; Figure 3B).

We further tested the efficiency of post-administration of RvD2 in existing cancer pain relief. In the von Frey test, a single delivery of RvD2 (i.t., 500 ng) on day 14 following sarcoma implantation (in the late phase) exhibited the quick and transitory mitigation of the established mechanical allodynia for 3 h (F (1, 50) = 27.97, *p* < 0.0001, *n* = 6, two-way ANOVA; Figure 3C) in the mice experiencing bone cancer. As a parallel, in the hotplate test, post-treatment with RvD2 also decreased the existing heat hyperalgesia for 3 h, as reflected by the increase in the thermal PWL in the bone cancer animals (F (1, 50) = 8.494, *p* = 0.0053, *n* = 6, two-way ANOVA; Figure 3D). Collectively, intrathecal RvD2 therapy confers protection against the development of bone cancer pain following sarcoma implantation.

### 3.4. Intrathecal Pre-Application of RvD2 Down-Modulates Spinal IL-17 and CXCL1 Overexpression and Astrocyte Activation after Sarcoma Cell Implantation

**Figure 4:**
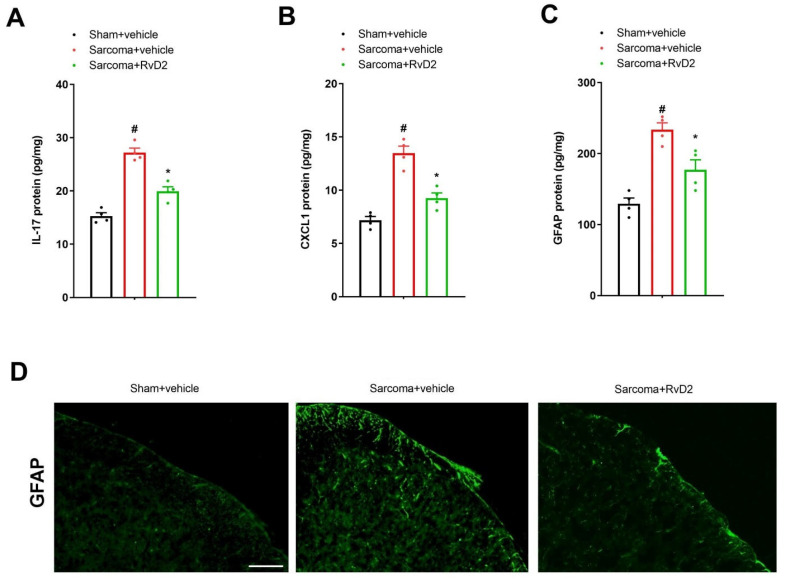
Pretreatment with RvD2 reduced spinal IL-17 secretion, CXCL1 release and astrocyte activation in animals with bone cancer pain. Intrathecal RvD2 (500 ng) was given daily on days 4, 5 and 6 following sarcoma cell implantation. All data from biochemical experiments were collected on day 7 following sham and sarcoma implantations. (**A**–**C**) ELISA assay showed that RvD2 pre-treatment down-modulated the elevated levels of spinal IL-17, CXCL1 and GFAP (the marker of astrocyte activation) proteins in sarcoma animals. (**D**) Immunohistochemistry staining showed representative photomicrographs of spinal GFAP following sarcoma interventions and RvD2 exposure (scale bar, 50 μm). Results from biochemical experiments are presented as the mean ± SEM (*n* = 4) and compared using one-way ANOVA followed by Bonferroni multiple comparisons. # *p* < 0.05 vs. group sham + vehicle. * *p* < 0.05 vs. group sarcoma + vehicle.

The spinal levels of IL-17, CXCL1 and GFAP were also measured to evaluate if these proinflammatory molecules were implicated in RvD2 anti-nociception in bone cancer pain. As noted, we observed an obvious elevation in the spinal expressions of IL-17, CXCL1 and GFAP in the animals 7 days after sarcoma cell implantation using an ELISA assay (*p* < 0.05, *n* = 4; Figure 4A–C). Interestingly, repetitive pre-administration of RvD2 (i.t., 500 ng) suppressed the overexpression of IL-17, CXCL1 as well as GFAP proteins in the dorsal horn following sarcoma exposure (*p* < 0.05, *n* = 4; Figure 4A–C). We also detected a reduction in sarcoma-induced spinal activation of astrocytes by RvD2 pretreatment in the immunohistochemistry experiments (Figure 4D). Taken together, these biochemical findings suggest that RvD2 prevents chronic bone cancer pain through spinal suppression of neuroinflammation.

### 3.5. Spinal Neutralization of IL-17 Alleviates Chronic Neuropathic Pain and Bone Cancer Pain by Reducing CXCL1 Expressions

**Figure 5:**
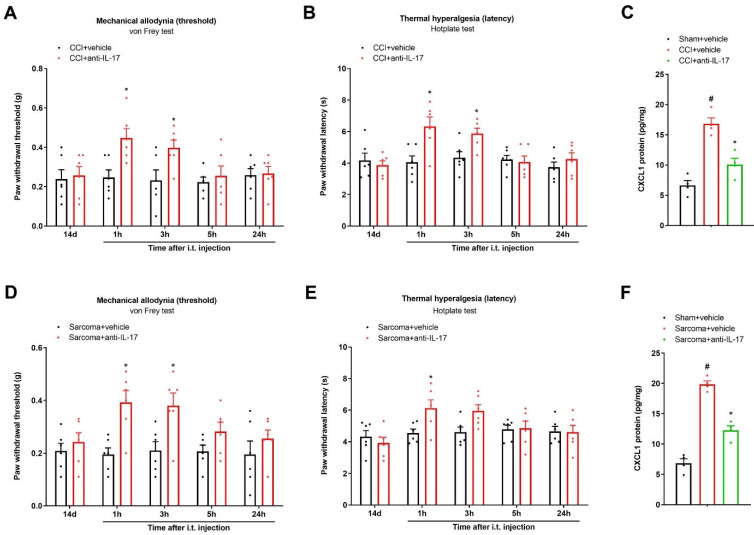
Spinal neutralization of IL-17 reduced chronic neuropathic pain and bone cancer pain. A neutralizing antibody against IL-17 (anti-IL-17) was intrathecally injected (2 μg) on day 14 after peripheral nerve injuries and sarcoma cell implantations, respectively. Mechanical allodynia (**A**) and thermal hyperalgesia (**B**) after CCI surgeries and anti-IL-17 injections were documented using von Frey experiments and hotplate experiments, respectively. (**C**) ELISA assay showed that anti-IL-17 administration reversed the elevated expressions of spinal CXCL1 proteins in CCI animals. # *p* < 0.05 vs. group sham + vehicle. * *p* < 0.05 vs. group CCI + vehicle. (**D**,**E**) Intrathecal injection of anti-IL-17 impaired the established bone cancer pain in sarcoma-treated mice. (**F**) ELISA assay showed that anti-IL-17 administration reversed the elevated expressions of spinal CXCL1 proteins in sarcoma animals. # *p* < 0.05 vs. group sham + vehicle. * *p* < 0.05 vs. group sarcoma + vehicle. Results from behavioral experiments are presented as the mean ± SEM (*n* = 6) and compared using two-way ANOVA followed by Bonferroni multiple comparisons. Results from biochemical experiments are presented as the mean ± SEM (*n* = 4) and compared using one-way ANOVA followed by Bonferroni multiple comparisons. i.t. = intrathecal injection.

To further evaluate whether the spinal IL-17 inflammatory cascade is important in the pathophysiology of long-lasting pain, anti-IL-17 (i.t., 2 μg) was injected once 14 days after peripheral nerve trauma. Strikingly, post-administration of anti-IL-17 exhibited a dramatic attenuation of mechanical allodynia and heat hyperalgesia, which was sustained for over 3 h, as reflected by the increase in the PWT (F (1, 50) = 9.887, *p* = 0.0028; *n* = 6, two-way ANOVA; Figure 5A) and the extension of the PWL (F (1, 50) = 9.461, *p* = 0.0034, *n* = 6, two-way ANOVA; Figure 5B) in the CCI mice. Meanwhile, an application of anti-IL-17 (i.t., 2 μg) on day 14 following sarcoma cell implantation ameliorated the existing mechanical allodynia (F (1, 50) = 20.96, *p* < 0.0001, *n* = 6, two-way ANOVA; Figure 5D) and thermal hyperalgesia (F (1, 50) = 4.404, *p* = 0.0409, *n* = 6, two-way ANOVA; Figure 5E). Moreover, the ELISA assay found that anti-IL-17 therapy downregulated spinal CXCL1 expression in the animals undergoing peripheral nerve trauma and sarcoma implantation (*p* < 0.05, *n* = 4; Figure 5C,F). Collectively, these behavioral findings reveal the involvement of spinal IL-17 and CXCL1 signaling in the pathogenesis of persistent bone cancer pain and neuropathic pain.

### 3.6. Intrathecal Administration of RvD2 Reverses IL-17-Caused Acute Pain and Rise in Spinal CXCL1 Levels

**Figure 6:**
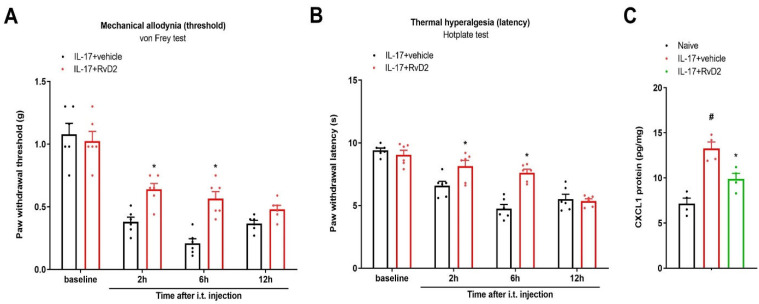
Generation of acute pain by IL-17 exposure was reduced after co-administration of RvD2. RvD2 (i.t., 500 ng) was administered 1 h prior to recombinant IL-17 application (i.t., 400 ng). Mechanical allodynia was evaluated by measuring paw withdrawal threshold (**A**) using von Frey experiments, and thermal hyperalgesia was evaluated by measuring paw withdrawal latency (**B**) using hotplate experiments. Exogenous IL-17-induced acute pain behaviors were ameliorated by co-application of RvD2. (**C**) ELISA assay showed that RvD2 administration inhibited the elevated expressions of spinal CXCL1 proteins in IL-17-treated animals. Behavioral results are presented as the mean ± SEM (*n* = 6) and compared using two-way ANOVA followed by Bonferroni multiple comparisons. Results from biochemical experiments are presented as the mean ± SEM (*n* = 4) and compared using one-way ANOVA followed by Bonferroni multiple comparisons. # *p* < 0.05 vs. group naïve. * *p* < 0.05 vs. group IL-17 + vehicle. i.t. = intrathecal injection.

Next, we explored the hypothesis that RvD2 reduces IL-17-mediated spinal pain perception. RvD2 (i.t., 500 ng) was administered 1 h prior to exogenous IL-17 application (i.t., 400 ng). Intriguingly, the von Frey test and hotplate test showed that RvD2 pretreatment reduced IL-17-associated mechanical allodynia (F (1, 40) = 19.55, *P* < 0.0001, *n* = 6, two-way ANOVA; Figure 6A) and heat hyperalgesia (F (1, 40) = 18.13, *p* = 0.0001, *n* = 6, two-way ANOVA; Figure 6B). More intriguingly, recombinant IL-17 delivery through the i.t. route elevated the spinal CXCL1 concentration, which could be compromised following RvD2 intervention (*p* < 0.05, *n* = 4; Figure 6C). Thus, the above specific findings further indicate that IL-17-dependent inflammatory cascades may be neuroprotective targets of RvD2 analgesia in nociception states.

### 3.7. Intrathecal Administration of RvD2 Reduces Exogenous CXCL1-Induced Acute Pain

**Figure 7:**
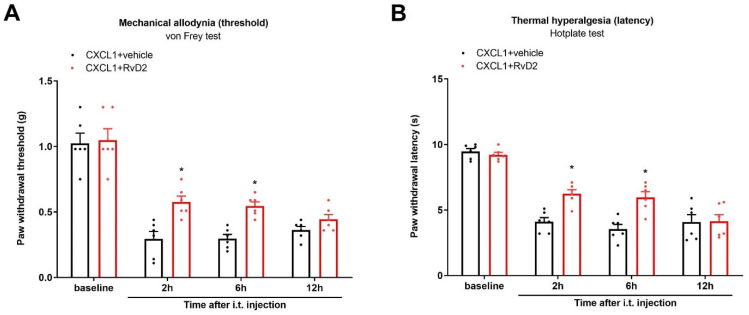
Generation of acute pain by CXCL1 exposure was reduced after co-administration of RvD2. RvD2 (i.t., 500 ng) was administered 1 h prior to recombinant CXCL1 application (i.t., 100 ng). Mechanical allodynia was evaluated by measuring paw withdrawal threshold (**A**) using von Frey experiments, and thermal hyperalgesia was evaluated by measuring paw withdrawal latency (**B**) using hotplate experiments. Exogenous CXCL1-caused acute nociceptive phenotypes were ameliorated by co-application of RvD2. Results are presented as the mean ± SEM (*n* = 6) and compared using two-way ANOVA followed by Bonferroni multiple comparisons. * *p* < 0.05 vs. group CXCL1 + vehicle. i.t. = intrathecal injection.

In addition, we investigated whether RvD2 controls CXCL1-mediated spinal pain transduction. RvD2 (i.t., 500 ng) was administered 1 h prior to recombinant CXCL1 application (i.t., 100 ng). Strikingly, behavioral experiments discovered that pre-injection of RvD2 reduced CXCL1-elicited mechanical allodynia (F (1, 40) = 17.47, *p* = 0.0002, *n* = 6, two-way ANOVA; Figure 7A) and thermal hyperalgesia (F (1, 40) = 16.4, *p* = 0.0003, *n* = 6, two-way ANOVA; Figure 7B). Thus, these specific findings further indicate the implication of CXCL1-dependent inflammatory cascades in RvD2 anti-nociception and neuroprotection in nociception states.

### 3.8. Systemic Therapy of RvD2 Alleviates Chronic Neuropathic Pain and Bone Cancer Pain

**Figure 8:**
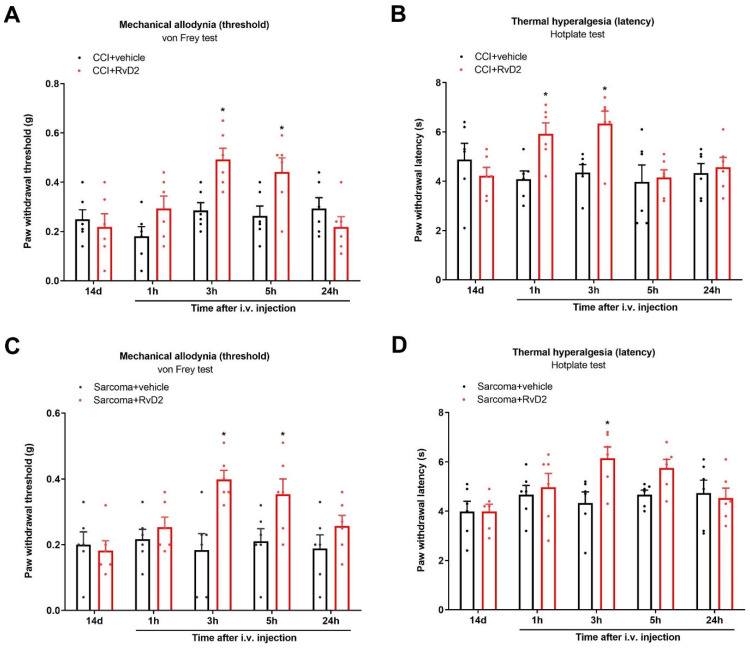
Systemic therapy of RvD2 reduced the existing chronic neuropathic pain and bone cancer pain. RvD2 (5 μg) was intravenously (i.v.) given on day 14 following peripheral nerve trauma and sarcoma cell implantation, respectively. Mechanical allodynia was evaluated by measuring paw withdrawal threshold (**A**) using von Frey experiments, and thermal hyperalgesia was evaluated by measuring paw withdrawal latency (**B**) using hotplate experiments following CCI surgeries and RvD2 administrations. * *p* < 0.05 vs. group CCI + vehicle. (**C**,**D**) RvD2 post-treatment attenuated the established persistent bone cancer pain. * *p* < 0.05 vs. group sarcoma + vehicle. Results from behavioral experiments are presented as the mean ± SEM (*n* = 6) and compared using two-way ANOVA followed by Bonferroni multiple comparisons.

Finally, to explore whether systemic RvD2 treatment is effective against pathological pain syndrome, we injected RvD2 (5 μg) following the i.v. route on day 14 following peripheral nerve trauma. Intriguingly, systemic (i.v.) treatment with RvD2 ameliorated the established CCI-caused mechanical allodynia and heat hyperalgesia, as identified by the rise in the mechanical PWT (F (1, 50) = 7.623, *p* = 0.008, *n* = 6, two-way ANOVA; Figure 8A) and the extension of the heat PWL (F (1, 50) = 6.062, *p* = 0.0173, *n* = 6, two-way ANOVA; Figure 8B). Meanwhile, a single injection of RvD2 (i.v., 5 μg) on day 14 following sarcoma cell implantation relieved the existing mechanical allodynia (F (1, 50) = 13.99, *p* = 0.0005, *n* = 6, two-way ANOVA; Figure 8C) as well as thermal hyper-nociception (F (1, 50) = 5.184, *p* = 0.0271, *n* = 6, two-way ANOVA; Figure 8D).

## 4. Discussion

In this present study, we discovered the following findings. First, intrathecal pre-administration of RvD2 prevented persistent mechanical allodynia and heat hyperalgesia after peripheral nerve trauma and sarcoma implantation. Second, intrathecal post-treatment with RvD2 relieved the established chronic neuropathic pain and bone cancer pain. Third, intrathecal RvD2 strategy reduced the up-modulation of IL-17 secretion, CXCL1 release and astrocyte activation in the dorsal horn in chronic pain states. Fourth, spinal IL-17 neutralization was effective against CCI-caused neuropathic pain and bone cancer pain through decreasing CXCL1’s release. Fifth, pre-administration of RvD2 impaired both recombinant IL-17-caused acute pain and exogenous CXCL1-caused acute pain. Sixth, systemic RvD2 therapy was sufficiently effective in attenuating mechanical and heat hypersensitivity in animals with peripheral nerve injuries and bone cancer. Therefore, all results recapitulated an unexplored property of RvD2 as a therapy of chronic pain alleviation by spinal suppression of astrocyte-dependent neuroinflammation.

Neuroinflammatory responses are considered key steps for chronic pain pathogenesis after osteoarthritis, fractures, peripheral nerve trauma, cancer and opioid medications [7,10,11]. The accumulating literature demonstrates that astrocytes serve as the main modulators of several neurological diseases and neuropsychiatric disorders, which may be through driving and maintaining neuroinflammation [13,14]. Importantly, the reduction in astrocyte proliferation and GFAP expression following nerve damage is sufficient to cause the behavioral attenuation of mechanical allodynia [32]. Recent reports also reveal that astrocyte-derived inflammatory mediators (cytokines and chemokines) in the dorsal horn of the spinal cord are tightly correlated with nociceptive synaptic transmission and pain perception [9,15]. For example, it has been identified that chemokine CCL2 and CXCL1 in astrocytes is required for neuroinflammation and neuronal plasticity in neuropathic pain states following injuries to peripheral nerves [33,34]. Moreover, intrathecal intervention with tumor necrosis factor-α (TNF-α)-activated astrocytes induces long-lasting mechanical allodynia through persistent generations of CCL2 and CXCL1 in the dorsal horn, suggesting the interaction of astrocytic cytokine and chemokine in pain initiation and chronification [34]. Given that IL-17 expressed by astrocytes is an important mediator of chronic pain syndromes [16], we examined the potential crosstalk of IL-17 and CXCL1 in pain behaviors in our rodent models. Here, we first reported the spinal rise in IL-17 secretion, CXCL1 release and GFAP expression in mice with peripheral nerve trauma and sarcoma implantation, consistent with nociceptive behaviors of long-lasting mechanical allodynia as well as heat hyperalgesia. This study, for the first time, discovered that spinal IL-17 neutralization reduces CCI-induced neuropathic pain and sarcoma-induced bone cancer pain through the impairment of CXCL1’s release. Additionally, intrathecal administration of exogeneous IL-17 evokes acute pain symptoms and facilitates astrocytic CXCL1 release in naïve animals. These behavioral and biochemical findings strongly suggest the identification of spinal IL-17 and CXCL1 cascades in persistent pain development following nerve damage and bone cancer, pointing to the possibility that mitigating astrocyte-mediated neuroinflammatory cascades may provide a promising neuro-therapeutic avenue for chronic nociception states.

D-series resolvins exhibit potent anti-neuroinflammation and neuroprotection in neurodegenerative, neurodevelopmental and neuropathic disorders, such as Parkinson’s disease [35], sevoflurane-induced cognitive decline [36] and brain injuries after subarachnoid hemorrhage [37]. Recent studies have highlighted that D-series resolvins (RvD1 and RvD5) control inflammation and pain phenotypes in animals with gouty arthritis [38], pancreatitis [39], irritative chemical-induced intraplantar injuries [24], tibial fractures with orthopedic surgeries [25], and paclitaxel-induced peripheral neuropathy [26]. Yet, the therapeutic role and targets of RvD2 in chronic pain remains unclear. To the best of our knowledge, our study, for the first time, revealed that repetitive i.t. exposure to RvD2 prevents persistent mechanical allodynia as well as heat hyper-nociception following trauma to the sciatic nerves and sarcoma cell implantation. We next observe the alleviation of the existing chronic neuropathic pain and bone cancer pain with a single application of RvD2 through the i.t. route. More strikingly, this is the first study in which pre-treatment with RvD2 downregulated spinal IL-17 secretion, CXCL1 release and astrocyte activation in both the CCI and bone cancer animals. Moreover, RvD2 exposure reduced exogenous IL-17-elicited acute inflammatory pain and the spinal release of CXCL1. Intravenous (systemic) treatment with RvD2 is able to protect against chronic pain behaviors associated with peripheral nerve trauma and cancer. Collectively, these specific findings emphasize that RvD2 therapy controls the production and persistence of chronic pain by mitigating IL-17-dependent CXCL1 inflammatory cascades and astrocyte activation in the dorsal horn of the spinal cord. Indeed, astrogliosis is a key driver of functional potentiation in excitatory nociceptive neurons through the interaction of CCL2 (in astrocytes) and its receptor CCR2 (in neurons) [9,15]. Accordingly, it will be interesting to test whether CCL2 cascades are also downstream contributors in the analgesia and neuroprotection of RvD2 therapy.

Apart from astrocytes, microglia also serve as an important source of inflammatory mediators. Spinal microgliosis-mediated neuroinflammation and synaptic plasticity are also considered primary steps for central sensitization in the induction of fracture-associated mechanical allodynia and chemotherapy-caused neuropathy [40,41,42]. Further experiments are thus warranted to explore whether microglia-derived cytokines and chemokines are implicated in RvD2 analgesia for pathological pain. One limitation is the failure to ascertain whether these positive results are generalizable to other persistent pain populations, such as visceral pain, trigeminal neuralgia and migraines. Another limitation is that we did not investigate the therapeutic potential of RvD2 for females, which should be tested in the future. Additionally, neuropathic pain is correlated with depression and anxiety, and it would be of great interest to explore the effect of RvD2 on depression and anxiety during pain conditions.

In summary, the current findings identify an unrecognized pharmacological property of RvD2 in preventing and attenuating chronic neuropathic pain and bone cancer pain by spinal inhibition of IL-17 secretion, CXCL1 release and astrocyte activation. These results unveil that RvD2 therapy and IL-17 neutralization may generate novel pharmaceutical approaches for neuroinflammation control and pain treatment.

## Data Availability

All data relevant to the study are included in the article for figures and supplemental figures. Data are available from the corresponding author upon reasonable request.

## Supplementary Materials

The following supporting information can be downloaded at: https://www.mdpi.com/article/10.3390/brainsci13010152/s1.

## Abbreviations

ANOVA = analysis of variance; CCI = chronic constriction injury; CFA = complete Freund’s adjuvant; DHA = docosahexaenoic acid; ELISA = enzyme-linked immunosorbent assay; EPA = eicosapentaenoic acid; GFAP = glial fibrillary acidic protein; IL-17 = interleukin-17; i.t. = intrathecal; i.v. = intravenous; PWT = paw withdrawal threshold; PWL = paw withdrawal latency; RvD2 = resolvin D2; SNL = spinal nerve ligation; SPMs = specialized pro-resolving mediators; TNF-α = tumor necrosis factor-α.

## References

[1] Cohen S.P., Vase L., Hooten W.M. (2021). Chronic pain: An update on burden, best practices, and new advances. Lancet.

[2] Mills S.E.E., Nicolson K.P., Smith B.H. (2019). Chronic pain: A review of its epidemiology and associated factors in population-based studies. Br. J. Anaesth..

[3] Descalzi G., Ikegami D., Ushijima T., Nestler E.J., Zachariou V., Narita M. (2015). Epigenetic mechanisms of chronic pain. Trends Neurosci..

[4] Yongjun Z., Tingjie Z., Xiaoqiu Y., Zhiying F., Feng Q., Guangke X., Jinfeng L., Fachuan N., Xiaohong J., Yanqing L. (2020). A survey of chronic pain in China. Libyan J. Med..

[5] Bliss T.V., Collingridge G.L., Kaang B.K., Zhuo M. (2016). Synaptic plasticity in the anterior cingulate cortex in acute and chronic pain. Nat. Rev. Neurosci..

[6] Li X.H., Chen Q.Y., Zhuo M. (2020). Neuronal Adenylyl Cyclase Targeting Central Plasticity for the Treatment of Chronic Pain. Neurotherapeutics.

[7] Baral P., Udit S., Chiu I.M. (2019). Pain and immunity: Implications for host defence. Nat. Rev. Immunol..

[8] Ji R.R., Chamessian A., Zhang Y.Q. (2016). Pain regulation by non-neuronal cells and inflammation. Science.

[9] Ji R.R., Donnelly C.R., Nedergaard M. (2019). Astrocytes in chronic pain and itch. Nat. Rev. Neurosci..

[10] Ji R.R., Nackley A., Huh Y., Terrando N., Maixner W. (2018). Neuroinflammation and Central Sensitization in Chronic and Widespread Pain. Anesthesiology.

[11] Ji R.R., Xu Z.Z., Gao Y.J. (2014). Neuroinflammation drives chronic pain: Emerging targets with pro- and anti-inflammatory roles. Nat. Rev. Drug Discov..

[12] Yang Q.Q., Zhou J.W. (2019). Neuroinflammation in the central nervous system: Symphony of glial cells. Glia.

[13] Hasel P., Rose I.V.L., Sadick J.S., Kim R.D., Liddelow S.A. (2021). Neuroinflammatory astrocyte subtypes in the mouse brain. Nat. Neurosci..

[14] Linnerbauer M., Wheeler M.A., Quintana F.J. (2020). Astrocyte Crosstalk in CNS Inflammation. Neuron.

[15] Donnelly C.R., Andriessen A.S., Chen G., Wang K., Jiang C., Maixner W., Ji R.R. (2020). Central Nervous System Targets: Glial Cell Mechanisms in Chronic Pain. Neurotherapeutics.

[16] Meng X., Zhang Y., Lao L., Saito R., Li A., Bäckman C.M., Berman B.M., Ren K., Wei P.K., Zhang R.X. (2013). Spinal interleukin-17 promotes thermal hyperalgesia and NMDA NR1 phosphorylation in an inflammatory pain rat model. Pain.

[17] Zhu C., Tang J., Ding T., Chen L., Wang W., Mei X.P., He X.T., Wang W., Zhang L.D., Dong Y.L. (2017). Neuron-restrictive silencer factor-mediated downregulation of μ-opioid receptor contributes to the reduced morphine analgesia in bone cancer pain. Pain.

[18] Liu X., Liu H., Xu S., Tang Z., Xia W., Cheng Z., Li W., Jin Y. (2016). Spinal translocator protein alleviates chronic neuropathic pain behavior and modulates spinal astrocyte-neuronal function in rats with L5 spinal nerve ligation model. Pain.

[19] Manjavachi M.N., Passos G.F., Trevisan G., Araújo S.B., Pontes J.P., Fernandes E.S., Costa R., Calixto J.B. (2019). Spinal blockage of CXCL1 and its receptor CXCR2 inhibits paclitaxel-induced peripheral neuropathy in mice. Neuropharmacology.

[20] Chiang N., Serhan C.N. (2020). Specialized pro-resolving mediator network: An update on production and actions. Essays Biochem..

[21] Serhan C.N., Levy B.D. (2018). Resolvins in inflammation: Emergence of the pro-resolving superfamily of mediators. J. Clin. Investig..

[22] Roh J., Go E.J., Park J.W., Kim Y.H., Park C.K. (2020). Resolvins: Potent Pain Inhibiting Lipid Mediators via Transient Receptor Potential Regulation. Front. Cell Dev. Biol..

[23] Wang Y.H., Gao X., Tang Y.R., Chen F.Q., Yu Y., Sun M.J., Li Y. (2022). Resolvin D1 Alleviates Mechanical Allodynia via ALX/FPR2 Receptor Targeted Nod-like Receptor Protein 3/Extracellular Signal-Related Kinase Signaling in a Neuropathic Pain Model. Neuroscience.

[24] Xu Z.Z., Zhang L., Liu T., Park J.Y., Berta T., Yang R., Serhan C.N., Ji R.R. (2010). Resolvins RvE1 and RvD1 attenuate inflammatory pain via central and peripheral actions. Nat. Med..

[25] Zhang L., Terrando N., Xu Z.Z., Bang S., Jordt S.E., Maixner W., Serhan C.N., Ji R.R. (2018). Distinct Analgesic Actions of DHA and DHA-Derived Specialized Pro-Resolving Mediators on Post-operative Pain after Bone Fracture in Mice. Front. Pharmacol..

[26] Luo X., Gu Y., Tao X., Serhan C.N., Ji R.R. (2019). Resolvin D5 Inhibits Neuropathic and Inflammatory Pain in Male but Not Female Mice: Distinct Actions of D-Series Resolvins in Chemotherapy-Induced Peripheral Neuropathy. Front. Pharmacol..

[27] Zhu M., Yuan S.T., Yu W.L., Jia L.L., Sun Y. (2017). CXCL13 regulates the trafficking of GluN2B-containing NMDA receptor via IL-17 in the development of remifentanil-induced hyperalgesia in rats. Neurosci. Lett..

[28] Donnelly C.R., Jiang C., Andreessen A.S., Wang K., Wang Z., Ding H., Zhao J., Luo X., Lee M.S., Lei Y.L. (2021). STING controls nociception via type I interferon signalling in sensory neurons. Nature.

[29] Shao S., Xu C.B., Chen C.J., Shi G.N., Guo Q.L., Zhou Y., Wei Y.Z., Wu L., Shi J.G., Zhang T.T. (2021). Divanillyl sulfone suppresses NLRP3 inflammasome activation via inducing mitophagy to ameliorate chronic neuropathic pain in mice. J. Neuroinflamm..

[30] Cui W., Li Y., Wang Z., Song C., Yu Y., Wang G., Li J., Wang C., Zhang L. (2021). Spinal caspase-6 regulates AMPA receptor trafficking and dendritic spine plasticity through netrin-1 in postoperative pain after orthopedic surgery for tibial fracture in mice. Pain.

[31] Zhang L., Zhao Y., Gao T., Zhang H., Li J., Wang G., Wang C., Li Y. (2022). Artesunate Reduces Remifentanil-induced Hyperalgesia and Peroxiredoxin-3 Hyperacetylation via Modulating Spinal Metabotropic Glutamate Receptor 5 in Rats. Neuroscience.

[32] Tsuda M., Kohro Y., Yano T., Tsujikawa T., Kitano J., Tozaki-Saitoh H., Koyanagi S., Ohdo S., Ji R.R., Salter M.W. (2011). JAK-STAT3 pathway regulates spinal astrocyte proliferation and neuropathic pain maintenance in rats. Brain.

[33] Chen G., Park C.K., Xie R.G., Berta T., Nedergaard M., Ji R.R. (2014). Connexin-43 induces chemokine release from spinal cord astrocytes to maintain late-phase neuropathic pain in mice. Brain.

[34] Gao Y.J., Cheng J.K., Zeng Q., Xu Z.Z., Decosterd I., Xu X., Ji R.R. (2009). Selective inhibition of JNK with a peptide inhibitor attenuates pain hypersensitivity and tumor growth in a mouse skin cancer pain model. Exp. Neurol..

[35] Krashia P., Cordella A., Nobili A., La Barbera L., Federici M., Leuti A., Campanelli F., Natale G., Marino G., Calabrese V. (2019). Blunting neuroinflammation with resolvin D1 prevents early pathology in a rat model of Parkinson’s disease. Nat. Commun..

[36] Sun Q., Yan H., Chen F., Jiang F., Chen W., Li D., Guo Y. (2021). Restoration of Proresolution Pathway with Exogenous Resolvin D1 Prevents Sevoflurane-Induced Cognitive Decline by Attenuating Neuroinflammation in the Hippocampus in Rats with Type 2 Diabetes Mellitus. Front. Pharmacol..

[37] Zhang T., Zuo G., Zhang H. (2022). GPR18 Agonist Resolvin D2 Reduces Early Brain Injury in a Rat Model of Subarachnoid Hemorrhage by Multiple Protective Mechanisms. Cell Mol. Neurobiol..

[38] Zaninelli T.H., Fattori V., Saraiva-Santos T., Badaro-Garcia S., Staurengo-Ferrari L., Andrade K.C., Artero N.A., Ferraz C.R., Bertozzi M.M., Rasquel-Oliveira F. (2022). RvD1 disrupts nociceptor neuron and macrophage activation and neuroimmune communication, reducing pain and inflammation in gouty arthritis in mice. Br. J. Pharmacol..

[39] Quan-Xin F., Fan F., Xiang-Ying F., Shu-Jun L., Shi-Qi W., Zhao-Xu L., Xu-Jie Z., Qing-Chuan Z., Wei W. (2012). Resolvin D1 reverses chronic pancreatitis-induced mechanical allodynia, phosphorylation of NMDA receptors, and cytokines expression in the thoracic spinal dorsal horn. BMC Gastroenterol..

[40] Hu L.Y., Zhou Y., Cui W.Q., Hu X.M., Du L.X., Mi W.L., Chu Y.X., Wu G.C., Wang Y.Q., Mao-Ying Q.L. (2018). Triggering receptor expressed on myeloid cells 2 (TREM2) dependent microglial activation promotes cisplatin-induced peripheral neuropathy in mice. Brain Behav. Immun..

[41] Zhang L., Li N., Zhang H., Wang Y., Gao T., Zhao Y., Wang G., Yu Y., Wang C., Li Y. (2022). Artesunate therapy alleviates fracture-associated chronic pain after orthopedic surgery by suppressing CCL21-dependent TREM2/DAP12 inflammatory signaling in mice. Front. Pharmacol..

[42] Chen G., Zhang Y.Q., Qadri Y.J., Serhan C.N., Ji R.R. (2018). Microglia in pain: Detrimental and protective roles in pathogenesis and resolution of pain. Neuron.

