# Supplementary figures and images for: Resolvin D2 Reduces Chronic Neuropathic Pain and Bone Cancer Pain via Spinal Inhibition of IL-17 Secretion, CXCL1 Release and Astrocyte Activation in Mice
Source: Brain Sci. 2023 Jan 15;13(1):152. doi: 10.3390/brainsci13010152 (PMC9856778; doi:10.3390/brainsci13010152)

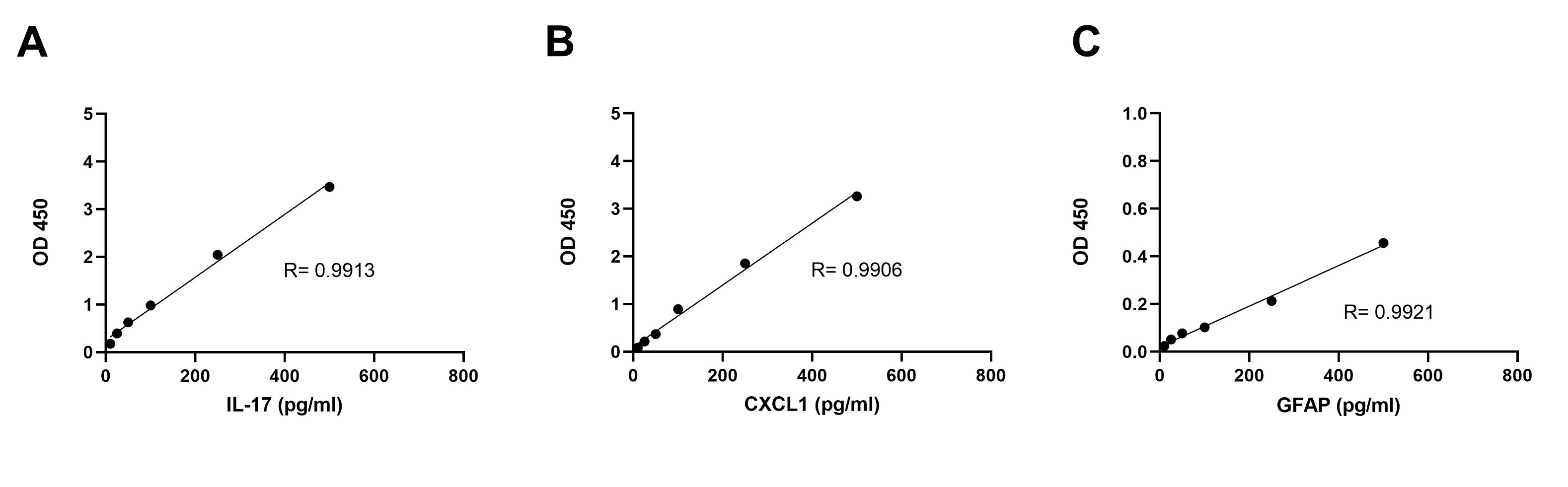

Supplement: Supplementary file 1 [file brainsci-13-00152-s001.zip › brainsci-2141713-supplementary.jpg]
